# A night on call or an overnight shift does not reduce residents’ empathy: a randomized crossover multicenter survey

**DOI:** 10.1186/s12909-019-1822-5

**Published:** 2019-10-26

**Authors:** Michiko Mizobe, Hitomi Kataoka, Hiroshi Yamagami, Chikao Ito, Yasuaki Koyama, Erika Yawata, Takashi Shiga

**Affiliations:** 1 Department of Emergency and Critical Care Medicine, Tokyo Bay Urayasu Ichikawa Medical Center, 3-4-32 Todaijima, Urayasu, Chiba 279-0001 Japan; 2 Department of Primary Care and Medical Education, Okayama University Medical School, 2-5-1 Shikata, Kita, Okayama, 700-8558 Japan; 3 Department of Emergency Medicine, Shonan Kamakura General Hospital, 1370-1 Okamoto, Kamakura, Kanagawa 247-8533 Japan; 4 Department of Emergency Medicine, Asahi General Hospital, I 1326, Asahi, Chiba 289-2511 Japan; 5 Department of Emergency and Critical Care Medicine, University of Tsukuba Hospital, 2-1-1 Amakubo, Tsukuba, Ibaraki 305-8576 Japan; 6 Emergency & Critical Care, Cardiovascular, Stroke Center Niigata City General Hospital, 463-7 Shumoku, Chuo, Niigata, Niigata, 950-1197 Japan; 7 Department of Emergency Medicine, International University of Health and Welfare School of Medicine, 1-4-3 Mita, Minato, Tokyo, 108-8329 Japan

**Keywords:** Empathy, Sleep deprivation, Night call, Overnight shift

## Abstract

**Background:**

Studies have shown that sleep deprivation may reduce empathy among medical students. Yet, little is known about the empathy after a night on call or an overnight shift among resident physicians. Hence, we aimed to examine whether a night on call or an overnight shift reduces the physicians’ empathy.

**Methods:**

We conducted a multicenter randomized crossover survey using the Jefferson Scale of Physician Empathy (JSE). A total of 260 physicians who worked at academic hospitals and community hospitals in Japan in 2016 were recruited and randomized into two groups. Group A first completed the JSE prior to a night on call or an overnight shift; then, 8 weeks later, Group A completed the JSE after a night on call or an overnight shift. Group B first completed the JSE after a night on call or an overnight shift; then, 8 weeks later, Group B completed the JSE prior to a night on call or an overnight shift. Statistical analyses were performed to compare the JSE scores of pre- and post-night on call or overnight shifts.

**Results:**

A total of 117 Group A physicians and 112 Group B physicians returned a completed JSE. The overall response rate was 88.08%. There was no significant difference in the JSE scores between pre- and post-night on call or overnight shift. (Group A before night vs Group B after night, *p* = 0.40, Group A after night vs Group B before night, *p* = 0.68).

**Conclusion:**

As per our results, a night on call or an overnight shift did not reduce the Japanese physicians’ empathy. To the best of our knowledge, this is the first study on physicians’ empathy after a night on call or an overnight shift.

## Background

Empathy is essential in achieving an optimal patient outcome. It represents the capacity of the physician to perceive the world from the patient’s perspective. Therefore, physicians need to be educated on the importance of empathy as an integral part of healthcare professionalism. Despite the consensus of professional organizations and medical education leaders on the importance of empathy, empirical research on the relation between sleep deprivation and the erosion of empathy is scarce. Guadagni reported that sleep deprivation had a negative effect on emotional empathy among healthy volunteers [1]. Hojat proposed that a significant decline in empathy occurs during the third year of medical school, when the curriculum shifts toward patient-care activities [2]. This decline in empathy is attributed to a high volume of learning materials and to sleep deprivation. A night on call or an overnight shift provides the physician with a challenging situation with high responsibility, which may affect the physician’s well-being, sleep quality, and fatigue and may therefore result in poor communication and patient complaints. During overwhelming on-call rotations or overnight shifts, physicians display lower attention and vigilance [3–6]. One study revealed that post-call performance showed impairments similar to those associated with a 0.04 to 0.05 g % blood alcohol concentration [4].

A night on call mostly starts from 5 pm after a daytime work and ends at 8 am–9 am time range. A night shift mostly starts from 5 pm without a preceding daytime work and ends at 8 am–9 am time range. Currently, most doctors work on call at night instead of overnight shift in Japan. Based on a previous Japanese study, sleep deprivation after a night on call is estimated to be sleep hour less than 4 h [7]. Unfortunately, in Japan, most of on-call doctors are not able to leave in the next morning, but must stay at the hospital to perform their usual daytime tasks. A large-scale survey indicated that 26.4% of Japanese residents worked more than 80 h a week [8]. Having a brief look at international situations, heavy on-call work might cause sleep deprivation [9], and a night shift work is characterized by increased sleepiness during the shift [10]. However, no empirical evidence correlates the postgraduate physicians’ empathy with sleep deprivation. To address this knowledge gap, we tested the hypothesis that the sleep deprivation caused by a night on call or an overnight shift diminishes physicians’ empathy. We performed a multicenter randomized crossover survey to examine the effect of a night on call or an overnight shift on physicians’ empathy.

## Methods

### Participants

Japanese residents who worked at Tokyo Bay Medical Center, Shonan Kamakura General Hospital, Asahi General Hospital, University of Tsukuba Hospital, and Niigata City General Hospital participated in this multicenter randomized crossover survey in 2016. The abovementioned hospitals are teaching hospitals that have both a junior residency program and a senior residency program. All hospitals are the tertiary care medical institutions (more than 300 beds). Niigata City Hospital is located in the northern part of Japan, while the other hospitals are located in the greater Tokyo area. University of Tsukuba Hospital is an academic medical center with a medical school. We asked transitional year residents, internal medicine residents and emergency medicine residents (post graduate years 1 to 6) who worked at each hospital to participate. They were explained the principles of the study and the residents who agreed to participate were assigned to randomization. Their participation was voluntary. Participants were informed that individual survey answers would be kept confidential and used for research purposes only.

### Instrument

Regarding the previous study on empathy, the empathy scale can be measured by using the Jefferson Scale of Physician Empathy (JSE) [2, 11–15]. The physician version (HP-Version) of the JSE used in this study included 20 items answered on a 7-point Likert-type scale ranging from 1 (strongly disagree) to 7 (strongly agree). Satisfactory evidence in support of the psychometric properties of this 7-point Likert-type scale has been reported [2, 11–15]. The JSE has been translated into 42 languages, including Japanese. The JSE was translated into Japanese by Kataoka using a back-translation procedure [11]. The JSE total score was calculated based on the responses to each item and the total score of JSE minus the corresponding items.

### Procedures

We numbered the participants and randomly divided the numbers into two groups by using a table of random digits. Group A physicians first answered the JSE prior to a night on call or an overnight shift first; then, 8 weeks later, physicians answered the JSE again after a night on call or an overnight shift. Group B physicians first answered the JSE after a night on call or an overnight shift; then, 8 weeks later, physicians answered the JSE again prior to a night on call or an overnight shift. We explained that the JSE evaluates physician’s empathy and that the results were for research purposes only. The survey was printed for distribution to each participant. Those surveys to which the participants could not respond because they were not working at the site or those with missing data for calculating JSE score were excluded from the study. The responses from participants were anonymous. This study was approved by the research ethics committee of each hospital. Physicians were not compensated for their participation in this study.

### Outcome measure

The primary outcome of interest was the difference in JSE scores between those completed prior to a night on call or an overnight shift and those complete after a night on call or an overnight shift.

### Statistical analyses

We calculated that the observation of 91 physicians in each group would provide 80% power to detect a 5% decrease in the JSE score (115 points pre vs. 110 points post). We compared the JSE scores of a pre- and post-night on call or overnight shift by using a Wilcoxon rank sum test. We used variance analysis to score differences in gender, age, postgraduate years, marital status, responsibility in child upbringing, and currently rotating department. A *p* value of < 0.05 was considered statistically significant. Statistical analyses were performed with SPSS version 22.

## Results

### Response rate

**Fig. 1:**
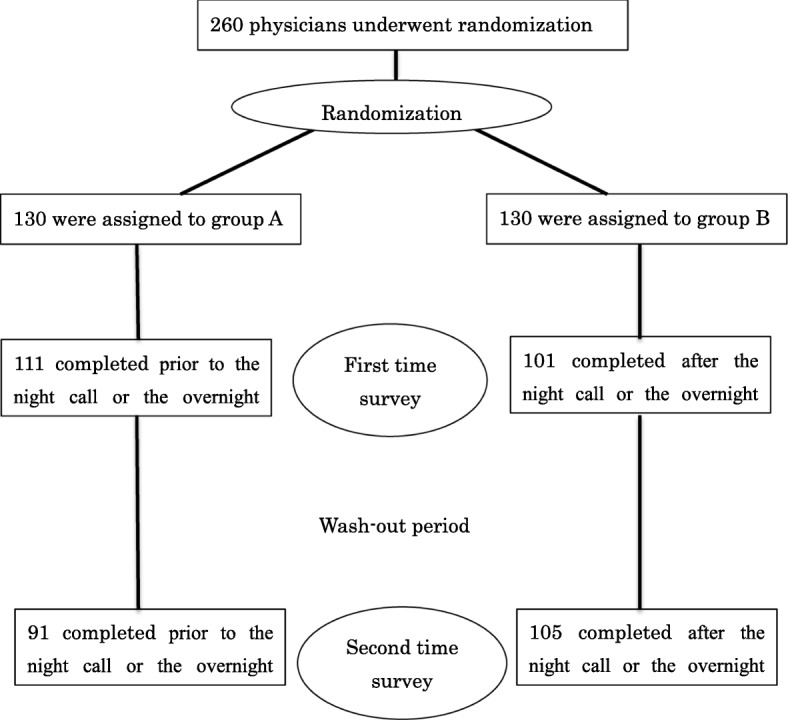
Participant flow

**Fig. 2:**
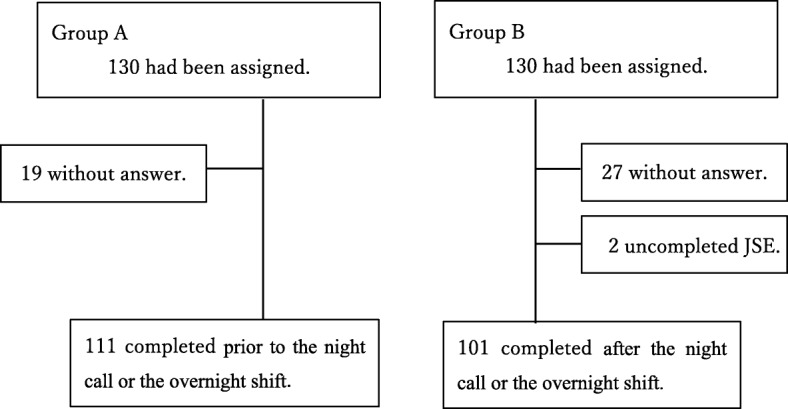
Participant flow at the first time

**Fig. 3:**
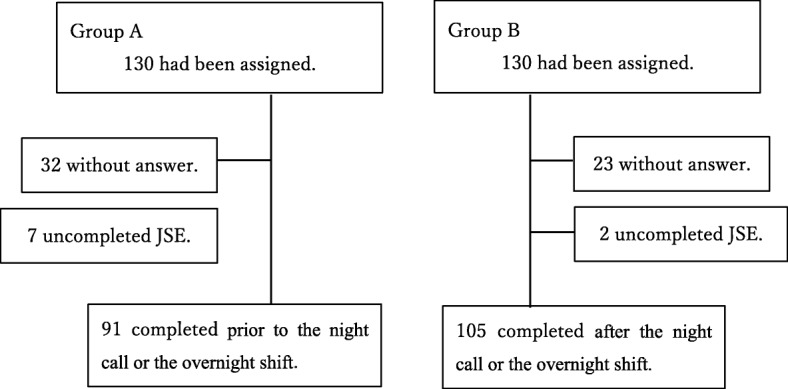
Participant flow at the second time

The JSE was given to 130 physicians in each group for empathy evaluation (Fig. 1). For the first time survey, 111 Group A physicians and 101 Group B physicians returned a completed JSE (Fig. 2). After the wash-out period of 8 weeks, for the second time survey, 91 Group A physicians and 105 Group B physicians returned a completed JSE (Fig. 3). The overall response rate was 88.08%. The response rate prior to the night on call or the overnight shift was 94.32%, and the response rate after the night on call or the overnight shift was 79.48%. There was a significant difference in the response rate to JSEs administered prior to a night on call or an overnight shift compared to those administered after the night on call or the overnight shift (*p* ≤ 0.01).

### Baseline characteristics

The characteristics of physicians who completed the JSE were not different between the two groups (Tables 1 and 2).

**Table 1:** Baseline characteristics of physicians

	Group A *n* = 117	Group B *n* = 112	*p* value
Male	89 (76.07%)	81 (72.32%)	0.31
Female	28 (23.93%)	19 (27.68%)	
Age > 30 years	18 (15.38%)	18 (16.07%)	0.95
Age ≤ 30 years	99 (84.62%)	94 (83.93%)	
Junior residents	72 (61.54%)	69 (61.61%)	0.88
Senior residents	45 (38.46%)	43 (38.39%)	
Married	21 (17.95%)	18 (16.07%)	0.64
Responsibility in child upbringing	6 (5.13%)	8 (7.14%)	0.52
Athlete club participation	79 (67.52%)	83 (74.11.%)	0.34

**Table 2:** Clinical rotation of the participants

	The first time survey			The second time survey		
	Group A	Group B	*p* value	Group A	Group B	*p* value
Internal Medicine	41 (36.94%)	42 (46.15%)	0.19	33 (36.26%)	48 (45.71%)	0.18
General Surgery	10 (9.01%)	9 (9.89%)	0.83	8 (8.79%)	11 (10.48%)	0.69
EM/ICU^a^	18 (16.21%)	7 (7.69%)	0.07	9 (9.89%)	12 (11.43%)	0.72
Pediatrics	3 (2.70%)	2 (2.20%)	1.00	3 (3.30%)	1 (0.95%)	0.34
Orthopedics	1 (0.90%)	1 (1.10%)	1.00	0 (0%)	1 (0.95%)	1.00
Neurosurgery	4 (3.60%)	0 (0%)	0.13	3 (3.30%)	0 (0%)	0.10
Anesthesiology	4 (3.60%)	4 (4.40%)	1.00	3 (3.30%)	3 (2.86%)	1.00
Obstetrics and Gynecology	8 (7.21%)	2 (2.20.%)	0.19	6 (6.59%)	3 (2.86%)	0.31
Psychiatry	3 (2.70%)	5 (5.49%)	0.47	3 (3.30%)	6 (5.71%)	0.51
Others	18 (16.21%)	17 (18.68%)	0.71	18 (19.78%)	18 (17.14%)	0.63
No answer	0 (0%)	3 (3.30%)	0.09	4 (4.40%)	2 (1.90%)	0.42

^a^*EM/ICU* emergency medicine and critical medicine

### Comparison of JSE total scores

The group JSE median scores and interquartile range are reported in Table 3. There was no significant difference in JSE scores between Group A’s pre-night on call or overnight shift results and Group B’s post-night on call or overnight shift results. Likewise, there was no significant difference in JSE scores between Group B’s pre-night on call or overnight shift results and Group A’s post-night on call or overnight shift results. A characteristics-based comparison of JSE scores did not show reduction in JSE total scores after a night on call or overnight shift (Table 4).

**Table 3:** The JSE^a^ scores

	Group A Pre-night on call or overnight shift	Group B Post-night on call or overnight shift	*p* value
Median score of JSE (IQR^b^)	110 (102–119)	108 (101–118)	0.40
	Group B Pre-night on call or overnight shift	Group A Post-night on call or overnight shift	*p* value
Median score of JSE (IQR^b^)	105 (99–117)	105 (97–116)	0.68

^a^*JSE* Jefferson Scale of Physician Empathy

^b^*IQR* interquartile range

**Table 4:** Characteristic-based comparison of JSE^a^ scores

Median (IQR^b^)	Pre-night on call or overnight shift	Post-night on call or overnight shift	*p* value
Sex			
Male	107 (99–118)	106 (97–117)	0.33
Female	107 (99–118)	106 (97–117)	0.33
Age			
> 30 years old	107 (102–117)	109 (102–117)	0.83
≤ 30 years old	109 (100–118)	107 (97–117)	0.29
Resident			
Junior	110 (101–119)	109 (99–118)	0.52
Senior	105 (100–116)	105 (101–115)	0.63
Marriage			
Single	109 (101–118)	108 (99–117)	0.57
Married	111 (102–120)	107 (101–118)	0.48
Responsibility in child upbringing			
Yes	112 (104–121)	106 (100–114)	0.20
No	108 (100–118)	108 (99–118)	0.66
Athlete club participation			
Yes	108 (100–119)	107 (98–118)	0.44
No	110 (102–117)	109 (102–117)	0.88

^a^*JSE* Jefferson Scale of Physician Empathy

^b^*IQR* interquartile range

Significantly higher scores of JSE in post-night on call or overnight shift were seen in physicians working rotational shifts in emergency medicine and critical medicine (EM/ICU) departments compared to those working in other departments. There was no significant difference, however, between the pre-night on call or overnight shift JSE scores for physicians working in EM/ICU and those working in other departments (Table 5).

**Table 5:** Clinical rotation-based comparison of JSE^a^ scores

JSE score	EM/ICU^b^	Other departments	*p* value
Pre-night on call or overnight shift n Median (IQR^c^)	30 115 (102–122)	186 108 (100–117)	0.08
Post-night on call or overnight shift n Median (IQR^c^)	16 117 (106–121)	166 107 (99–116)	0.02

^a^*JSE* Jefferson Scale of Physician Empathy

^b^*EM/ICU* emergency medicine and critical medicine

^c^*IQR* interquartile range

## Discussion

In this multicenter randomized crossover survey, we found that one night on call or an overnight shift did not reduce the Japanese physicians’ empathy. To the best of our knowledge, this is the first study to reveal that physicians’ empathy doesn’t lessen despite of a night on call or an overnight shift. Several factors can explain these findings.

First, the physicians who participated in this study were relatively young, approximately 75% of them were less than 30 years old. In the Japanese medical education curriculum, most physicians start learning about healthcare professionalism and communications skills after they start their residency; moreover, it is found that young physicians lack concern about healthcare professionalism or communication skills during medical schools [16, 17]. Most of this sturdy’s participants of this study were in the middle of their training and had developed concerns about healthcare professionalism and communication skills; therefore, their empathy may have been sufficiently high enough to compensate for the exhaustion that follows a night on-call or an overnight shift.

Second, we measured empathy following only one night on-call or overnight shift. Some of the participants may have been working frequent night shifts and some may not have been. In other words, we did not measure the cumulative fatigue following several night shifts, which may have contributed to the results.

The higher JSE scores were found in physicians who worked rotational shifts in EM/ICU department, which can be explained by the unique features of EM/ICU rotations. First, most Japanese EM/ICU departments have adopted shift-work systems, whereas the other departments have not. The shift-work system can cause sleep deprivation because it affects the circadian rhythm; however, this is not as serious as the sleep deprivation caused by the on-call system. EM/ICU physicians are able to focus on their work without much concern about sleep deprivation. The second unique feature of EM/ICU work is that physicians encounter new clinically ill cases during every shift and they are required to focus fully on the patient’s care and make appropriate treatment decisions promptly. Furthermore, physicians often face patient’s deaths and their families’ grief, and these emotional events may have a strong impact on the physicians’ feelings. Third, clinical rotation in EM/ICU educates the physicians on patient intensive care and motivates them, which makes them prone to empathize with patients in each case.

A previous study revealed that medical students’ empathy declined in the later years of medical school, which was mainly attributed to a workload increase and sleep deprivation [2]. However, despite our hypothesis, we found that empathy did not decline after a night on call or an overnight shift in this study. There are several reasons for this. The first reason is the cultural feature of Japan. The Japanese values maintaining harmonious relationships with others despite their fatigue, and this sense of virtue motivates the Japanese physicians to maintain their empathy. The second reason is the differential physicians’ status. In previous studies, the participants were medical students [2], whereas we surveyed physicians in this study. Physicians have more responsibility in managing patients than do students, and they can manage their tasks independently, saving their efforts during their nights on call.

Response bias is one of the limitations in this study. The physicians who have reduced empathy might not be able to put effort into answering this survey. If those surveys were included, the influence of night shift may have decreased the empathy we found in our study. Second, there is a possibility of discordance between residents’ attitudes and actual behaviors. The participants might have reported their answers in order to be socially-accepted, ideal doctor. While self-reported questionnaires have limitations, this offers an important first step toward an understanding of the motivations underlying possible actions. Third, while we calculated the sample size prior to survey administration, the sample size could be a limitation. A broader sample size might offer a different result. Fourth, the population in our study was relatively young. There is a study that reveals elder volunteers over the age of 55 years were able to bear a single night of acute sleep deprivation remarkably well [18]. Fifth, our cohort comprised a mixture of post-on-call and overnight shift physicians, and the results could be heterogeneous.

According to our study design, we incorporated an eight-week interval between the two JSE surveys to eliminate the influence of the first survey. However, influential events that alter a physician’s empathy, such as emotional life events or cumulative fatigue, may have occurred during the eight-week interval. We were unable to eliminate such influences in this study.

We did not measure the number of sleeping hours during the on-call night or overnight shifts and did not focus on the frequency of on-call night or overnight shifts. According to previous studies, sleep deprivation during an overnight shift, intervals between each shift, or frequent night on call or overnight shifts are important factors of fatigue [6, 19]. Fatigue is one of the causes of reduced empathy in physicians.

## Conclusion

Our results showed that a night on call or an overnight shift did not reduce the Japanese physicians’ empathy. Further, higher empathy was seen in physicians working rotational shifts in EM/ICU after a night on call or an overnight shift compared to those working a night on call or an overnight shift in other departments.

## Data Availability

There are restrictions on the availability of data due to consent agreements for data security and IRB approval, which allow access only to the external researchers for research monitoring purposes.

## Abbreviation

JSE: Jefferson scale of physician empathy
